# Clinical and epidemiologic characteristics of hospitalized oncological patients with hypercalcemia: a longitudinal, multicenter study

**DOI:** 10.1007/s10354-024-01051-x

**Published:** 2024-07-23

**Authors:** Guillermo Ropero-Luis, Jaime Sanz-Cánovas, Almudena López-Sampalo, Alberto Ruiz-Cantero, Ricardo Gómez-Huelgas

**Affiliations:** 1 Department of Internal Medicine, Hospital de la Serranía, Carretera Ronda-San Pedro km. 2, 29400 Ronda, Málaga, Spain; 2 Faculty of Medicine, University of Málaga, Boulevard Louis Pasteur 32, 29071 Málaga, Spain; 3 Department of Internal Medicine, Hospital Regional Universitario de Málaga, Avenida Carlos Haya s/n, 29010 Málaga, Spain; 4 Instituto de Investigación Biomédica de Málaga (IBIMA—Plataforma BIONAND), Málaga, Spain; 5 Physiopathology of Obesity and Nutrition Networking Biomedical Research Centre (CIBEROBN), Instituto de Salud Carlos III, 28029 Madrid, Spain

**Keywords:** Hypercalcemia, Calcium metabolism disorders, Paraneoplastic syndromes, Bisphosphonates, Calcitonin

## Abstract

**Background:**

There are few studies that have analyzed the characteristics of hypercalcemia in hospitalized oncological patients. Our objectives were to describe the clinical characteristics of hospitalized patients with paraneoplastic hypercalcemia and to identify prognostic variables for mortality.

**Methods:**

This was an observational, longitudinal, retrospective, and bicentric study. It included adult patients admitted to two hospitals in Málaga, Spain (2014–2018). The minimum follow-up period was 2 years or until death.

**Results:**

A total of 154 patients were included; the majority (71.4%) were admitted to the internal medicine department. The median follow-up was 3.5 weeks (interquartile range [IQR] 1.1–11.5). The mean (standard deviation) age was 67.6 (12.3) years, with a predominance of males (58.4%). The median (IQR) serum calcium at admission was 13.2 (11.8–14.6) mg/dl. The most common neoplasms were pulmonary (27.3%), hematologic (23.4%), urological (13%), and breast (12.3%). Furthermore, 56.5% of cases had a known history of neoplasia at the time of diagnosis. The parathyroid hormone (PTH) level was determined in 24%; of these, 10.8% had elevated levels. In all, 95.5% of patients died during follow-up. The median survival was 3.4 weeks (95% confidence interval 2.6–4.3). Factors associated with higher mortality were age, serum calcium at admission, previous history of neoplasia, etiology other than multiple myeloma, and noncorrection of hypercalcemia.

**Conclusions:**

In hospitalized patients, paraneoplastic hypercalcemia was associated with high short-term mortality. Several factors associated with a worse prognosis were identified in these patients.

## Introduction

Hypercalcemia, defined as a plasma calcium concentration equal to or greater than 10.2 mg/dl (2.5 mmol/l), arises from an imbalance in calcium entry into plasma, its elimination through urine, and its deposition in bone tissue. Various types of neoplasms, both solid and hematological, possess the capability to induce paraneoplastic (or tumoral) hypercalcemia through diverse mechanisms [1]. The predominant mechanism associated with this phenomenon is the production of parathyroid hormone-related peptide (PTHrP), with osteolytic metastases also contributing, albeit to a lesser extent. Other less frequent causes encompass hyperproduction of calcitriol, the presence of ectopic parathyroid hormone (PTH), and the release of cytokines that stimulate bone resorption. Clinically, hypercalcemia can manifest across a spectrum, ranging from asymptomatic cases to severe manifestations impacting the neurological and cardiovascular systems [2, 3].

Currently, there is a dearth of published studies examining the clinical and epidemiological characteristics of oncological patients presenting with hypercalcemia. Most available studies date back to the 20th century and fail to reflect subsequent scientific and medical advancements. Furthermore, information regarding the long-term prognosis of patients with paraneoplastic hypercalcemia is scarce and necessitates further in-depth investigation. This underscores the imperative for specific research addressing these issues within the context of contemporary medical care.

The primary aim of our study was to delineate the clinical and epidemiological characteristics of hospitalized oncological patients with hypercalcemia within a cohort treated at hospitals in Málaga. As secondary objectives, we sought to analyze potential prognostic variables for mortality among these patients.

## Materials and methods

### Study design, location, and participants

The study we conducted was observational, longitudinal, and retrospective. It was undertaken across two hospital centers: the Hospital Regional Universitario de Málaga (HRUM), which caters to a population of approximately one million inhabitants and housed 1017 beds in 2018, and the Hospital de la Serranía de Ronda (HSR), serving as the regional referral hospital for the Serranía de Málaga Health Management Area, with an assigned population of around 80,000 inhabitants and 152 beds in 2018.

Our target population comprised patients aged 18 and above who were hospitalized at HRUM and HSR between 2014 and 2018. We specifically selected patients with a documented diagnosis of hypercalcemia in their hospital discharge reports, utilizing the ICD-9-CM code 275.42 for the years 2014–2015 and the ICD-10-ES code E83.52 for the years 2016–2018. Patient data were sourced from the respective clinical documentation services. Notably, for this particular study, only cases of hypercalcemia associated with neoplastic processes were analyzed; the general characteristics of the cohort were outlined in a prior publication [4]. In instances where a patient had multiple admissions, data pertaining to the initial admission with such a diagnosis were gathered. Patient follow-up extended for a minimum of 2 years or until their death, with the end date of follow-up being December 31, 2020.

### Variables and data sources

We collected relevant epidemiological, clinical, and laboratory variables associated with the diagnosis and treatment of hypercalcemia, as well as the oncological processes of the patients within the cohort. For variables concerning blood calcium levels and creatinine, data from the initial laboratory test conducted at hospital admission were documented, along with subsequent tests indicating normalization of calcium levels, or alternatively, the last available test during the hospitalization period. Hypercalcemia was defined as plasma calcium levels equal to or exceeding 10.2 mg/dl, utilizing central laboratory values as a reference. Specific corrections were applied to account for the effects of albumin, or if unavailable, total proteins on calcium levels. Hypocalcemia (also corrected) was defined as plasma calcium levels below 8.5 mg/dl. Throughout data collection, commonly used formulas in clinical practice were employed to calculate corrected calcium [5, 6].

Based on plasma calcium levels, hypercalcemia was categorized as mild (10.2–11.9 mg/dl), moderate (12–13.9 mg/dl), or severe (≥ 14 mg/dl). In addition, clinical symptoms and signs upon admission were assessed, leading to the classification of patients into the following groups: asymptomatic, mild (marked by symptoms such as constipation, asthenia, or depressive mood), moderate (presenting symptoms like muscular weakness, lethargy, or neuropsychiatric alterations), and severe (exhibiting symptoms including stupor, coma, electrocardiographic alterations, or cardiac arrhythmias). PTH levels were classified based on reference values from hospital laboratories as low (< 15 pg/ml), normal (15–85 pg/ml), or high (> 85 pg/ml). The same criteria were applied to classify calcidiol levels as deficient (< 20 ng/ml), insufficient (20–30 ng/ml), adequate (30–150 ng/ml), or excessive (> 150 ng/ml). Data were manually extracted from the electronic medical records of the Andalusian Public Health System (Diraya), utilizing medical reports, laboratory tests, and prescription orders to compile available information.

### Statistical methods

Qualitative variables were presented using absolute and relative frequencies to depict the distribution of categories in each variable. Conversely, for continuous quantitative variables, they were reported as mean accompanied by standard deviation (SD) or, if deemed inappropriate, as median together with interquartile range (IQR). Prior to conducting parametric analyses, it was verified whether continuous quantitative variables adhered to a normal distribution through the Kolmogorov-Smirnov test. A significance level (α) of 0.05 was set for all statistical tests, and two-tailed tests were performed. In instances of missing values, they were excluded from the corresponding analyses to preserve result integrity. To assess the existence of statistically significant associations between qualitative and quantitative variables, specific tests such as the Mann–Whitney U or Kruskal–Wallis tests were applied, contingent upon the nature of the variables. Survival periods were expressed as median in weeks alongside their 95% confidence interval (CI). Survival analysis relied on robust statistical methods including the logarithmic method, Kaplan–Meier method, and Cox regression to evaluate and model patient survival, as well as explore variables potentially influencing this outcome. Statistical analysis was conducted using SPSS® Statistics 26 software (IBM, Armonk, NY, USA).

### Ethical aspects

The study received approval from the Provincial Research Ethics Committee of Málaga, with the waiver of obtaining informed consent from subjects justified for design reasons.

## Results

### General characteristics of the cohort

Of the 205 hospitalized patients who had documented hypercalcemia, 154 patients (75.1%) presented with tumoral hypercalcemia. The median follow-up of patients was 3.5 weeks (IQR 1.1–11.5), with a maximum of 279 weeks (5 years and 4 months). The general characteristics of this group of patients are detailed in Table 1. The average age of the patients was 67.6 years with a standard deviation (SD) of 12.3 years. In addition, 58.4% of the studied population were males. Regarding the distribution by medical services, most included patients had been attended to in the internal medicine service (71.4%), followed by oncology (15.6%), hematology (3.9%), urology (3.2%), pulmonology (2.6%), and other medical services (3.2%).

**Table 1:** Clinical–epidemiological characteristics of the patients included in the cohort

Age (years), mean (SD)	67.6 (12.3)	
Gender, *n* (%)	Male	90 (58.4)
	Female	64 (41.6)
Calcemia on admission (mg/dl), median (IQR)	13.2 (11.8–14.6)	
Symptoms at admission, *n* (%)	Asymptomatic	17 (11)
	Mild	51 (33.1)
	Moderate	83 (53.9)
	Severe	3 (1.9)
Severity of hypercalcemia, *n* (%)	Mild	44 (28.6)
	Moderate	60 (39)
	Severe	50 (32.5)
Chronology of malignancy, *n* (%)	Known	87 (56.5)
	New diagnosis	67 (43.5)
Type of malignancy, *n* (%)	Lung	42 (27.3)
	Multiple myeloma	24 (15.6)
	Urological	20 (13)
	Breast	19 (12.3)
	Hematologic (except MM)	12 (7.8)
	Otorhinolaryngological	11 (7.1)
	Digestive tract	8 (5.2)
	Hepatobiliopancreatic	5 (3.2)
	Gynecologic (except breast)	5 (3.2)
	Melanoma	3 (1.9)
	Unknown	5 (3.2)
Overall mortality, *n* (%)	147 (95.5)	

*SD* standard deviation, *IQR* interquartile range, *MM* multiple myeloma

The median calcium level at admission was 13.2 mg/dl (IQR 11.8–14.6). In most cases (87.9%), correction was performed using albumin levels. Based on calcium levels, patients were classified as mild, moderate, and severe in 28.5, 39, and 32.5% of cases, respectively. Based on clinical presentation at admission, patients were classified as asymptomatic, mild, moderate, and severe in 11, 33.1, 53.9, and 1.9%, respectively.

At the time of hypercalcemia diagnosis, 56.5% of the cases had a known history of neoplasia, while in the remaining 43.5%, neoplasia was discovered during the study of hypercalcemia. The most frequent types of neoplasms were pulmonary (27.3%), multiple myeloma (15.6%), urological (13%), and breast (12.3%). A total of 66.7% of cases had some type of bone metastasis, but no statistically significant association with calcium levels was found.

**Fig. 1:**
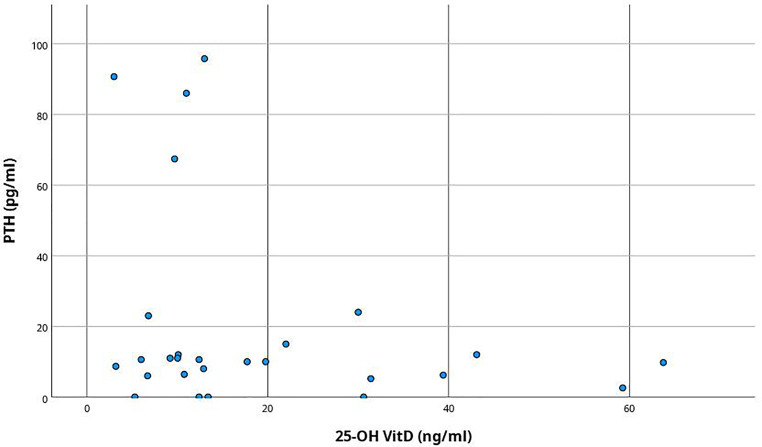
Dot plot comparing the distribution of parathyroid hormone (PTH) and 25-hydroxyvitamin D (25-OH VitD) values in oncology patients in the cohort who had these determinations available

Regarding biochemical analysis, PTH levels were requested in 24% (*n* = 42) and calcidiol levels in 22.7% (*n* = 40) of cases. Among patients with these determinations, 75.7% had low PTH levels, 13.5% had levels close to the lower limit of normal (less than 30 pg/ml), and 10.8% had high levels. Among the 4 patients with unsuppressed PTH levels, 2 were diagnosed with multiple myeloma, 1 patient had lung neoplasia and 1 had otorhinolaryngological neoplasia. Their calcium levels at admission ranged between 11 and 12.3 mg/dl. All of them had calcidiol levels in the insufficient range (below 20 ng/ml, Fig. 1). Three of these patients had a newly diagnosed neoplasm.

The occurrence of acute renal failure (ARF), analyzed in 136 patients, was found in 29.4% of cases. Regarding hypercalcemia treatment, a total of 145 patients were evaluated. Most of them (87.5%) received multiple hypocalcemic therapies as part of their management with the most commonly used being fluid therapy (89.1%), bisphosphonates (69.2%), loop diuretics (69.2%), corticosteroids (53.1%), and calcitonin (7.5%). The hypercalcemia correction rate was 62.8%; the median time until correction was 5 days (IQR 3–10). In addition, 45.3% of patients developed hypocalcemia.

### Survival analysis

**Fig. 2:**
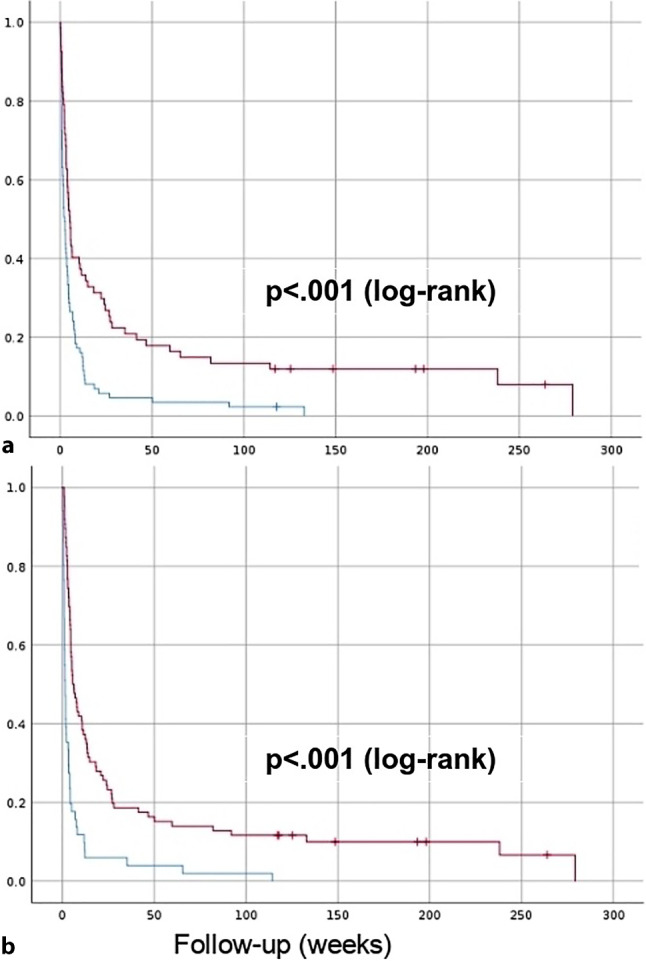
Survival functions. (**a**) The group of patients with newly diagnosed neoplasms (*red, upper*) and of the group with previous history of neoplasm (*blue, lower*). (**b**) The group of patients with corrected tumor hypercalcemia (*red, upper*) and of the group with uncorrected tumor hypercalcemia (*blue, lower*)

In all, 95.5% of the patients deceased during follow-up. The median survival was 3.4 weeks (95% confidence interval [CI] 2.6–4.3). Median survival differed significantly (*p* < 0.001) between patients with a new neoplasm diagnosis (5.1 weeks; 95% CI 3.7–6.6) compared to those with a previous history (2.4 weeks; 95% CI 1.4–3.4). Similarly, median survival was significantly higher (*p* < 0.001) in patients with corrected hypercalcemia (5.6 weeks; 95% CI 3–8.2) compared to those in whom it was not corrected (1.4 weeks; 95% CI 0.9–2). Survival functions are depicted in Fig. 2.

Median survival significantly varied (*p* < 0.001) between patients with multiple myeloma (24.4 weeks; 95% CI 0–52.4) compared to other patients (2.9 weeks; 95% CI 1.9–3.8). Remarkably, only 4 patients survived during follow-up with neoplasms other than multiple myeloma: 1 with urological neoplasia, 1 with melanoma, 1 with breast neoplasia, and 1 with hematological neoplasia.

A multivariate regression model was constructed to examine the effects of different variables on the probability of death during follow-up. Statistically significant associations were found with age (higher probability with increasing age), calcium level at admission (higher probability with higher calcium), previous neoplasia history (higher probability), etiology other than multiple myeloma (higher probability), and hypercalcemia correction (higher probability if not corrected). No statistically significant associations were found with gender, number of treatments administered, or presence of bone metastases. Results of the analysis are summarized in Table 2 (variables without statistically significant association are not included).

**Table 2:** Multivariate regression model to study the effects of different variables on the probability of death from any cause during patient follow-up (only analyses with statistically significant results are shown)

	HR	95% CI	*p*
Etiology other than myeloma	3	1.7–5.3	< 0.001
Previous history of malignancy	1.9	1.3–2.8	0.001
Calcemia at admission (mg/dl)	1.12	1.01–1.23	0.024
Age (years)	1.03	1.01–1.04	0.001
Corrected hypercalcemia	0.53	0.35–0.8	0.003

*HR* hazard ratio, *CI* confidence interval

### Comparative analysis between patients with previous neoplasia history and new diagnosis

The mean age in patients with a previous history of neoplasia was 65.5 years (SD 13) compared to 70.3 years (SD 11) in patients with a new diagnosis, with this difference being statistically significant (*p* = 0.02). The distribution of different types of neoplasia in both groups is shown in Table 3. In patients with a previous history of neoplasia, the median time from the initial neoplasia diagnosis to hypercalcemia detection was 22 weeks (IQR 6–48). No statistically significant differences were found in calcium levels at admission between the two groups.

**Table 3:** Distribution of the different neoplasms according to the time of diagnosis of hypercalcemia

Type of malignancy	Patients with prior history (%)	Newly diagnosed patients (%)
Lung	26.4	28.4
Breast	18.4	4.5
Urological	13.8	11.9
Otorhinolaryngological	10.3	3
Multiple myeloma	8	25.4
Digestive tract	5.7	4.5
Hematologic (except MM)	4.6	11.9
Hepatobiliopancreatic	4.6	–
Gynecologic (except breast)	4.6	–
Other	3	10.5

*MM*: multiple myeloma

Patients with a new diagnosis had a significantly higher hypercalcemia correction rate (72.6 vs. 54.7%; *p* = 0.03). Mortality during follow-up was significantly higher in the group with a previous history of neoplasia (98.9 vs. 91%; *p* = 0.04), which is also reflected in a lower overall survival (Fig. 2a).

## Discussion

This study on hospitalized patients with paraneoplastic hypercalcemia represents the first conducted in Spain to date. However, it carries certain limitations. First, direct subject follow-up was not conducted, relying instead on data available in the electronic medical records. Second, case selection depended on the diagnosis coding in the discharge report, potentially leading to the exclusion of some patients. Despite these limitations, the main strength of the study lies in its longitudinal and multicenter nature.

We opted to gather albumin-corrected calcium values, deemed more reliable [6], or alternatively, total protein-corrected values, as they align with common practice in routine clinical settings. In cases where both corrections were feasible, significant differences (> 0.5 mg/dl) were observed in half of the instances, predominantly favoring albumin correction. The practice of calcium correction by albumin remains controversial, as noted in prior the literature [7, 8], but certain studies have demonstrated its utility in oncologic patients [9]. Ideally, the utilization of ionized calcium values would have been preferred [10], but unfortunately, they were not routinely determined.

Patients with paraneoplastic hypercalcemia exhibited sociodemographic characteristics and calcium levels at admission similar to those of non-oncologic patients in our series [4]. The majority of patients were attended by internal medicine services, underscoring the crucial role of internists in their management.

It is noteworthy that PTH and calcidiol levels were requested in only about a quarter of cases, a trend consistent with other series such as that published by Balentine et al. [11], where PTH levels were requested in only 31% of 10,432 patients with hypercalcemia. Presumably, in many cases, the etiologies were apparent, rendering further investigation unnecessary. However, some studies have revealed that up to a third of oncologic patients with hypercalcemia actually had nonparaneoplastic causes [12]. In our cohort, 10.8% of oncologic patients with PTH determination had elevated levels, suggesting the possibility of undiagnosed primary hyperparathyroidism, although the concurrent presence of vitamin D deficiency precludes definitive assertions.

Mortality was nearly universal, and the median survival did not extend beyond a month. Survival was significantly higher in patients with newly diagnosed neoplasms, although the difference scarcely exceeded 3 weeks. This dismal short-term prognosis associated with the diagnosis of paraneoplastic hypercalcemia was consistent across all types of neoplasms, except for multiple myeloma, which exhibited a median survival approaching 6 months. Two older series of patients with paraneoplastic hypercalcemia reported median survivals ranging between 1–2 months and 1 year survivals between 6.3–31% [13, 14]. Despite therapeutic advances over the past 30 years, the prognosis of patients with paraneoplastic hypercalcemia appears to remain as dire as it was three decades ago.

In the multivariate analysis, calcium levels at admission, age, and hypercalcemia correction demonstrated associations with mortality. Patients with neoplasms other than multiple myeloma exhibited a threefold higher risk of death. In addition, the risk of death for patients with a previous history of neoplasia was double that of patients with a new diagnosis, likely due to the generally more advanced or aggressive nature of their neoplasms.

It is noteworthy that patients with a previous history of neoplasia were, on average, 5 years younger at the time of hypercalcemia detection compared to patients with a new diagnosis. Furthermore, patients with a previous history had a lower success rate in hypercalcemia correction and higher mortality during follow-up, suggesting that they likely suffered from more advanced or aggressive neoplasms.

## Conclusions

The presence of hypercalcemia in hospitalized oncologic patients signifies a dismal short-term prognosis, except in the case of patients with multiple myeloma, who exhibited higher survival rates. These findings underscore the critical importance of effectively managing hypercalcemia in oncologic patients to ensure adequate symptomatic control. Moreover, they highlight the necessity of providing palliative care to these patients to enhance their quality of life and alleviate suffering.
